# Rationale and Methods for Updated Guidelines for the Management of Penetrating Traumatic Brain Injury

**DOI:** 10.1089/neur.2022.0008

**Published:** 2022-06-21

**Authors:** Gregory W.J. Hawryluk, Shelley Selph, Angela Lumba-Brown, Annette M. Totten, Jamshid Ghajar, Bizhan Aarabi, James Ecklund, Stacy Shackelford, Britton Adams, David Adelson, Rocco A. Armonda, John Benjamin, Darrell Boone, David Brody, Bradley Dengler, Anthony Figaji, Gerald Grant, Odette Harris, Alan Hoffer, Ryan Kitigawa, Kerry Latham, Christopher Neal, David O. Okonkwo, Dylan Pannell, Jeffrey V. Rosenfeld, Guy Rosenthal, Andres Rubiano, Deborah M. Stein, Martina Stippler, Max Talbot, Alex Valadka, David W. Wright, Shelton Davis, Randy Bell

**Affiliations:** ^1^ Department of Neurosurgery, University of Manitoba, Winnipeg, Manitoba, Canada.; ^2^ Department of Medical Informatics and Clinical Epidemiology, Pacific Northwest Evidence-based Practice Center, Oregon Health & Science University, Portland, Oregon, USA.; ^3^ Department of Emergency Medicine, Stanford University School of Medicine, Stanford University, Palo Alto, California, USA.; ^4^ Stanford Neuroscience Health Center, Stanford University School of Medicine, Stanford University, Palo Alto, California, USA.; ^5^ University of Maryland Neurosurgery Associates, R Adams Cowley Shock Trauma Center, Baltimore, Maryland, USA.; ^6^ Inova Neuroscience and Spine Institute, Fairfax, Virginia, USA.; ^7^ Joint Trauma System, Department of Defense, Center of Excellence for Trauma, Baltimore, Maryland, USA.; ^8^ Independent Duty Medical Technician (IDMT), Hurlburt Field, Florida, USA.; ^9^ Barrow Neurological Institute at Phoenix Children's Hospital, University of Arizona College of Medicine, Phoenix, Arizona, USA.; ^10^ Department of Neurosurgery, MedStar Georgetown University Hospital, Washington, DC, USA.; ^11^ Anaethesia and Critical Care, Uniformed Services University, Bethesda, Maryland, USA.; ^12^ Department of Surgery, Memorial University of Newfoundland, St. John's, Newfoundland, Canada.; ^13^ Center for Neuroscience and Regenerative Medicine, Uniformed Services University, Bethesda, Maryland, USA.; ^14^ Department of Neurosurgery, Uniformed Services University, Bethesda, Maryland, USA.; ^15^ Department of Neurosurgery, University of Cape Town, Cape Town, Western Cape, South Africa.; ^16^ Department of Neurosurgery, Duke University, Raleigh, North Carolina, USA.; ^17^ Department of Neurosurgery, Stanford University School of Medicine, Stanford University, Palo Alto, California, USA.; ^18^ Department of Neurosurgery, Case Western Reserve University, Cleveland, Ohio, USA.; ^19^ McGovern Medical School, University of Texas, Houston, Texas, USA.; ^20^ Adult Outpatient Behavioral Health, Bethesda, Maryland, USA.; ^21^ Department of Neurosurgery Walter Reed National Military Medical Center, Bethesda, Maryland, USA.; ^22^ Department of Neurological Surgery, University of Pittsburgh, Pittsburgh, Pennsylvania, USA.; ^23^ Department of Surgery, University of Toronto, Toronto, Ontario, Canada.; ^24^ Department of Neurosurgery, The Alfred Hospital, Melbourne, Victoria, Australia.; ^25^ Hadassah University Medical Center, Jerusalem, Israel.; ^26^ INUB-Meditech Research Group, Neuroscience Institute, Universidad El Bosque, Bogota, Colombia.; ^27^ University of Maryland School of Medicine, Baltimore, Maryland, USA.; ^28^ Department of Neurosurgery, Beth Israel Deaconess Medical Center, Harvard Medical School, Boston, Massachusetts, USA.; ^29^ Royal Canadian Medical Service, Canadian Armed Forces, Canadian Forces Base Borden, Ontario, Canada.; ^30^ Department of Neurological Surgery, UT Southwestern Medical Center, Dallas, Texas, USA.; ^31^ Department of Emergency Medicine, Emory University, Atlanta, Georgia, USA.; ^32^ Department of Physical Medicine and Rehabilitation, Walter Reed National Military Medical Center, Bethesda, Maryland, USA.

**Keywords:** blast injury, guidelines, head trauma, penetrating brain injury, traumatic brain injury

## Abstract

Penetrating traumatic brain injury (pTBI) affects civilian and military populations resulting in significant morbidity, mortality, and healthcare costs. No up-to-date and evidence-based guidelines exist to assist modern medical and surgical management of these complex injuries. A preliminary literature search revealed a need for updated guidelines, supported by the Brain Trauma Foundation. Methodologists experienced in TBI guidelines were recruited to support project development alongside two cochairs and a diverse steering committee. An expert multi-disciplinary workgroup was established and vetted to inform key clinical questions, to perform an evidence review and the development of recommendations relevant to pTBI. The methodological approach for the project was finalized. The development of up-to-date evidence- and consensus-based clinical care guidelines and algorithms for pTBI will provide critical guidance to care providers in the pre-hospital and emergent, medical, and surgical settings.

## Introduction

Penetrating (pTBI) traumatic brain injury (TBI) is a catastrophic primary wounding mechanism encountered in military and civilian settings and is characterized by the violation of the skull and brain by a foreign body. For those who survive, the constellation of cerebral injuries in the setting of either pTBI or blast-related pTBI creates complex management scenarios for pre-hospital and in-hospital healthcare providers. Recovery and rehabilitation are often protracted and complicated for these patients.

Civilian pTBI accounts for an estimated 20,000 deaths in the United States each year with mortality in up to 90% of victims.^1,2^ The high lethality of civilian pTBI likely relates to the predominance of missile injury, including gunshot wounds, the absence of protective gear, and the violent circumstances that frequently surround the injury (i.e., attempted suicide, homicide, and other close-range mechanisms). From the military perspective, pTBI was prevalent in recent conflicts in Iraq and Afghanistan and most often results from a blast mechanism. pTBI outcomes in these modern conflicts are better than in past reports,^3–10^ most likely because of more aggressive clinical care.^6^ Before the conflicts in Iraq and Afghanistan, management in those who survived to reach care included limited surgical debridement and wound closure.^11^ In contrast, the new military model includes early cranial decompression and prevention of cerebrospinal fluid leak with skull base repair, causes of secondary brain injury are aggressively sought and mitigated (i.e., cerebrovascular injury), and a multi-disciplinary approach to cranial reconstruction is used.

pTBI clinical practice guidelines were first published in 2001 by an independent group.^12^ Topics addressed included antibiotic prophylaxis, antiseizure prophylaxis, management of cerebrospinal fluid leaks, surgical management, vascular complications, intracranial pressure monitoring, and prognosis. The 2001 guideline has not, however, been updated to reflect modern military experiences nor incorporate newer evidence- and consensus-based practices. There are no known published pTBI treatment algorithms informing patient management, despite the tremendous popularity of blunt TBI management algorithms^13–15^ and despite important changes to medical and surgical care over the past two decades. In response, the Brain Trauma Foundation (BTF) in collaboration with military and civilian TBI experts in the field, and with funding from the American Department of Defense (US Army Medical Research and Acquisition Command, BA200139: The Development of Best Practice Penetrating TBI Guidelines for Military and Civilian Patients) will generate an updated, evidence- and consensus-based pTBI clinical practice guideline and new care algorithms. The BTF has published numerous clinical practice guidelines for TBI care over the past quarter century^16,17^ and is undertaking this update with permission from the original pTBI guideline authors.

## Methods

### Key definitions

For this work, pTBI will be defined as a head injury with violation of, at minimum, the skull and likely dura and brain by a foreign body (Table 1). Linear non-displaced skull fractures alone, which result from a relevant wounding mechanism, will not be included. pTBI encompasses penetrating, tangential, and perforating injuries. pTBI has previously been subclassified as high-velocity missile, low-velocity missile, and non-missile injuries. However, because of the practical difficulty distinguishing high- and low-velocity missile injuries clinically, we will instead subclassify pTBI mechanistically as: 1) missile injuries (e.g., gunshot wounds); 2) blast fragment injuries (i.e., type II blast injury—blast overpressure plus fragment injury); and 3) low-velocity injuries (e.g., knives). Our working group ratified the latter classification scheme with a blinded consensus vote.

**Table 1.** Key Definitions

• **Penetrating traumatic brain injury (pTBI):** a head injury with violation of, at minimum, the skull and likely dura and brain by a foreign body. *Linear non-displaced skull fractures alone will NOT be included.*
• pTBI encompasses penetrating, tangential, and perforating injuries as follows:
○ **Penetrating:** a foreign object penetrates skull and dura and remains within the skull. This wounding mechanism lacks an exit wound.
○ **Tangential:** a foreign object glances off the skull, which often drives skull fracture fragments into the brain.
○ **Perforating:** a “through-and through” injury, characterized by entry and exit wounds. Of these three, perforating brain injuries are associated with a worse outcome.
• pTBI will be subclassified as:
○ **Missile injuries** (such as gunshot wounds)
○ **Blast fragment injuries** (such as type II blast injury—blast overpressure plus fragment injury)
○ **Low-velocity injuries** (such as knives)

### Development of an expert workgroup

BTF leadership, alongside civilian and military clinicians with topical expertise in pTBI, comprise the pTBI Expert Workgroup. The workgroup and its efforts are led by two cochairs with guidance and governance provided by a core multi-disciplinary steering committee. Workgroup members were selected for diversity among disciplinary expertise relevant to the management of TBI, scientific expertise, and demographic and geographical representation. Represented medical disciplines include neurosurgery, plastic surgery, neurology, general/trauma surgery, orthopedic surgery, ENT (ear, nose, and throat) surgery as well as emergency medicine, physical medicine, pediatrics, pre-hospital care, and rehabilitation. A military medic and a patient (service member recovered from a pTBI) are included. Sex and ethnic diversity was also specifically sought.

### Systematic review

#### Topic identification and refinement

Workgroup members were divided into three areas of clinical focus: pre-hospital and emergent management; surgical management; and medical management, led by A.L.B., B.A., and J.G., respectively. Each workgroup will update the topics included in the 2001 guidelines and will develop clinically relevant key questions as well as inclusion and exclusion criteria using the Population, Interventions, Comparators, Outcomes, Timing, Setting and Study design (PICOTS) framework. The present effort will endeavor to only inform aspects of care in which pTBI care would be distinct from, or otherwise insufficiently informed by, the “umbrella” recommendations found in the fourth edition of the Severe Traumatic Brain Injury Guidelines, which largely focus on blunt head injury.

Generated recommendations aim to be relevant to all patients with pTBI. However, evidence supporting distinct recommendations for key patient subgroups will be reported and may include differentiation by wounding mechanism (missile, blast fragment, or low-velocity injuries), military versus civilian victims, and adult versus pediatric victims.

#### Literature search

We will search Ovid MEDLINE, EMBASE, and Cochrane CENTRAL without date limits. The search strategy will be developed and executed by a specialized medical research librarian with experience in systematic reviews with peer review by a second librarian.

#### Study selection

Review of abstracts and full-text articles will be informed by pre-specified PICOTS and corresponding inclusion and exclusion criteria. All abstracts identified by the literature search will be assessed by one reviewer and excluded abstracts confirmed by a second reviewer. Independent, dual review of the full text of any potentially relevant article identified at abstract level will be conducted. Disagreements will be resolved by consensus or with the addition of a third reviewer. Included and excluded articles, and reasons for exclusion, at the full-text level will be provided to the Guideline Panel for review and will be included as an appendix in the full Evidence report.

Studies meeting inclusion criteria will be in children, adolescents, and/or adults with traumatic injuries that violate the skull and that provide evidence for a key question. Included outcomes of intervention studies consist of mortality, morbidity, function, and selected intermediate outcomes (Table 2). We will not exclude studies based on sample size, location of study, or baseline Glasgow Coma Scale score.

**Table 2.** Inclusion/Exclusion Criteria for Source Literature

PICOTS	Inclusion	Exclusion
Populations	All ages Penetrating brain injury including blast, tangential, and perforating injury from missiles, blast fragments, or low velocity • All baseline GCS levels • Mixed types of brain injury with at least 85% penetrating if results not reported individually	• Non-human studies • Injury limited to linear, non-displaced skull fracture or isolated face/neck injuries • Mixed types of brain injury and results not presented by injury type or <85% penetrating injury
Interventions	**Pre-hospital, prolonged field care, transport, ED, and trauma center evaluation:** • Initial resuscitation and prevention/mitigation of secondary injury • On-scene wound management • Cervical spine immobilization **Surgical management:** • Prevention and treatment of cerebrospinal fluid leaks • Vascular injuries • Foreign body removal and prerequisites • Cranial decompression **Medical management:** • Intracranial pressure monitoring (if distinct from fourth edition) • Intracranial pressure treatment (hyperosmolar therapy, lumbar drainage, CPP management including BP thresholds, ventilation therapies) • Seizure prophylaxis • Antibiotic prophylaxis • DVT prophylaxis • Chemoprophylaxis for stroke prevention	
Comparators	Placebo, no intervention, active control, waitlist control, delayed treatment, head-to-head studies	None
Outcomes	• Mortality • Neurological function • Selected morbidity • Cost	• Satisfaction • Quality of life • Sleep
Timing	Outcomes up to 1 year post-injury	Outcomes >1 year post-injury
Setting	Pre-hospital, trauma center, medical, surgical setting in all countries. Battlefield and mass causality as well as civilian	• Rehabilitation setting
Study designs	• All experimental study designs, observational studies including case series and case report • Epidemiological studies for salvageability • Current, well-conducted systematic reviews (may also use systematic reviews to identify studies searches may have missed)	• Abstracts, comments letters • Non-English language • Narrative reviews • Systematic reviews not meeting inclusion criteria

BP, blood pressure; CPP, cerebral perfusion pressure; DVT, deep vein thrombosis; GCS, Glasgow Coma Scale.

#### Data abstraction and risk of bias assessment

We will abstract data from all studies that include population characteristics (e.g., age, sex, wounding mechanism, and military/civilian status), intervention and comparator characteristics (e.g., methods of resuscitation, prophylactic drug and dose, and type of surgery performed), numbers enrolled and analyzed, relevant outcomes (i.e., mortality, neurological function, selected morbidities, and cost), and funding source.

All included studies will be assessed for risk of bias using criteria specific for the type of study design. Randomized trials will be assessed for risk of bias based on the randomization process, method for allocation concealment, similarity of baseline characteristics, blinding, missing data overall and differences between groups, whether intent-to-treat analysis was used, and possible reporting bias. Assessment of non-randomized studies with a comparison group will include selection of participants, whether differences in prognostic factors between groups were present, missing data, pre-specification of outcomes, whether ascertainment of outcomes was unbiased, and adjustment for potential confounding. Assessment of single-group trials will include selection of participants, pre-specification of outcomes, whether ascertainment of outcomes was unbiased, and assessment of missing data. Case series and case reports are studies with high risk of bias and will not be individually assessed. Study risk of bias will be assessed by two independent reviewers.

#### Data synthesis and quality of the body of evidence

When there is more than one study of an intervention, data will be synthesized quantitatively if a meta-analysis is appropriate (i.e., when studies are clinically homogeneous enough to provide meaningful combined estimates). When statistical heterogeneity is present in a pooled analysis, we will explore the reasons for this using stratified analysis and sensitivity analysis, as appropriate. We will also consider the potential effects of various participant subgroups (e.g., based on demographic characteristics, severity of injury, and injury mechanism) on intervention effects. When data cannot be pooled, we will provide a qualitative summary and analysis of findings.

**Table 3.** Proposed Topics for pTBI Evidence-Based Guidelines

• Overview: definitions, scope, and methodology
Pre-hospital
• Pre-hospital/emergent and prolonged field care, transport, and initial evaluation
• Prognosis and outcome prediction^a^
• Antibiotic prophylaxis: indications and agents
• Invasive and non-invasive neuroimaging: role, timing, and techniques
Surgical
• Indications, timing, and techniques for surgical management
• Vascular injuries: screening and management^a^
• Prerequisites for foreign body removal^a^
• Cerebrospinal fluid leaks: prevention, diagnosis, and management
Critical care
• Delayed vascular complications: screening and management including chemoprophylaxis for stroke prevention^a^
• Seizure prophylaxis: indications and agents
• Intracranial pressure monitoring

^a^ Denotes a new topic NOT previously included in published guidelines.^12^

pTBI, penetrating traumatic brain injury.

We will assess the quality of the body of evidence by outcome using the GRADE (Grading of Recommendations Assessment, Development and Evaluation) approach with the following criteria: risk of bias of included studies, consistency of effect, precision of the estimate, directness of the evidence, and potential publication bias.^9^ The quality of the body of evidence will be rated high, moderate, low, and very low. As an example, the highest-quality evidence (rated high) would come from multiple, well-conducted randomized trials with consistent findings, a precise pooled estimate of effect of the intervention on an included outcome, and be absent other bias. The lowest-quality evidence (rated very low) would come from expert opinion in the absence of any study data. We will generate a summary of findings table that includes ratings for quality of the body of evidence.

#### Development of recommendations

The development of recommendations is the next step after the evidence has been identified and synthesized and rated for risk of bias. Evidence-based recommendations are based on the quality of the body of evidence, applicability, and generalizability. For topics where there is little or no research, recommendations may be developed using rigorous Delphi consensus methodology.

Recommendations will be assigned a level based on the quality of the body of evidence. We will recognize five levels of recommendation based on the quality of the body of evidence:

- Level I recommendations are based on high quality of the body of evidence.
- Level II recommendations are based on moderate quality of the body of evidence.
- Level III recommendations are based on low quality of the body of evidence.
- Level IV recommendations are based on very low quality of the body of evidence.
- Level C: recommendations based on consensus in the absence of research evidence.

### Delphi process for consensus

Blinded consensus voting will be conducted to establish consensus for key aspects of the project. This will be particularly important for consensus-based algorithm development. A vote of 80% will be required to declare consensus as having been achieved where at least 80% of the panel participates in the vote. This is the same threshold used for consensus in the recent Seattle International Severe Traumatic Brain Injury Consensus Conference (SIBICC) effort.^14,18^ A week-long in-person meeting will be held 18 months into the 2-year project to finalize the recommendations and algorithm. A private company will facilitate electronic, blinded voting at this meeting to enable the Delphi methodology.

## Discussion

### Benefit from guideline and treatment algorithms

Clinical practice guidelines evaluate and consolidate available literature into evidence-based recommendations designed to inform best care. Guidelines for the management of TBI created by the BTF were the first surgical clinical practice guidelines published,^16^ and their implementation has been credited with an ∼50% reduction in mortality.^19,20^ Because of their demonstrated benefit, compliance with the BTF guidelines is mandated for U.S. trauma centers to maintain trauma accreditation.

Because of the success of the BTF guidelines and their demonstrated benefit, multiple editions of the BTF adult guidelines have been published,^15,16,21,22^ and TBI guidelines have been developed for numerous additional topics, including pediatrics,^23^ combat, pre-hospital care,^24^ and prognosis.^25^ The BTF's infrastructure has been key for disseminating and updating these important documents, with the most recent fourth edition TBI guidelines largely addressing closed head injury published in 2017.^22^

Algorithms for care bridge published evidence and its gaps with the realities of practice. Such care pathways were included with the first and second editions of the BTF adult guidelines, and they were the most popular aspect of these documents. These algorithms are inherently and necessarily consensus based, and their construction requires methodologies distinct from those for developing evidence-based clinical practice guidelines. To address the gap between evidence-based recommendations and patient care for severe blunt TBI patients, the SIBICC was recently convened, which led to two well-received and high-impact publications.^13–16,26^ In contrast, no such algorithms for the management of penetrating and blast-penetrating TBI have ever been published.

### Penetrating traumatic brain injury guidelines

Guidelines for the management of pTBI were last published in the *Journal of Trauma* more than two decades ago^12,27–33^ and, of course, do not reflect important subsequent advances in the literature and clinical standards of care (see *Literature Search* below).^34^ The combination of outdated pTBI guidelines and non-existent treatment algorithms may negatively impact morbidity and mortality because of the lack of consolidated clinician guidance on best practices for the medical and surgical care of pTBI. Because BTF did not lead the 2001 guideline, the organization sought permission to update the pTBI guidelines from the leadership of the original effort and this was granted.

### New penetrating traumatic brain injury literature

To estimate the volume of new studies to be screened and assist resource planning, a preliminary literature search was conducted (on April 8, 2020), using PubMed (https://www.ncbi.nlm.nih.gov/pubmed) and the basic search term “penetrating brain injury.” A total of 968 references were identified when limiting the search criteria to publication dates between January 1, 1933 and December 31, 2001. This search time frame includes the references that formed the foundation of the existing and outdated pTBI guidelines referenced above and specifically includes all periods of armed conflict from World War II to 2001. When using the same search term and limiting publication dates from January 1, 2002 to the present, 2413 candidate references were identified. This suggests that the overwhelming majority of the existing information on pTBI has been published after the last guidelines were generated in 2001 and, in large part, reflect the experiences and care provided during the conflicts in Iraq and Afghanistan. This affirms the strong need for updated pTBI guidelines.

### Contrasting civilian and military experiences

Although pTBI is problematic in both civilian and military populations, military neurosurgeons garner more experience managing these patients than most civilian neurosurgeons. Consequently, the military experience has led to important paradigm shifts in pTBI management over time. Such an important early advance for pTBI came from the Vietnam War. In the early days of the Vietnam War, neurosurgeons performed aggressive debridement of intracranial foreign bodies in an effort to prevent infection.^35–38^ A paradigm shift emanated from this conflict because it was judged that the neurological damage resulting from this aggressive debridement did more harm than good. As a result, to this day neurosurgeons typically leave inaccessible fragments behind at the time of an initial craniotomy, retrieving them later only if they should become infected.

The most recent conflicts in the Middle East have seen the longest period of sustained conflict in America's history. A return to more aggressive surgical and clinical approaches have seen outcomes in service members improve, beyond those typical of civilians despite more severe wounding mechanisms.^39^ The modern military paradigm involves early cranial decompression, prevention of cerebrospinal fluid leak with skull base repair, and aggressive efforts to mitigate secondary insults. One important study supporting this new approach involved wounded American soldiers from 2003 to 2008.^4^ Of the 408 head injuries studied, 228 were penetrating, with >80% resulting from primary and secondary blast injury. Of those who survived their initial trauma, field resuscitation, and transport to Walter Reed National Military Medical Center for definitive care, 154 received emergent decompressive craniectomy. A remarkably high proportion of these persons survived with good clinical outcomes.^4^ Increased attention to neurovascular injuries and vasospasms related to blast exposure has also been central to this new paradigm.^39–41^ These advances in military pTBI care are the central impetus for updating the pTBI guidelines.

### Blast as a factor complicating penetrating brain injury

The recent conflicts in Iraq and Afghanistan have created renewed interest in blast as a mechanism of TBI. The Centers for Disease Control and Prevention (CDC) has defined blast injuries as “…characterized by the anatomic or physiologic changes from the direct or reflective over-pressurization force impacting the body's surface.”^42^ Blast injuries are subdivided into four submechanisms: impact of body surfaces with the overpressure wave (primary blast injury); penetrating injury resulting from fragments propelled by blast (secondary blast injury); results of persons propelled or thrown by the blast wind (tertiary blast injury); and all explosion-related injuries (e.g., burns, crush injury, and quaternary blast injury). Given the importance of blast injury accompanying pTBI in the military, we have decided to include an analysis of such patients in this project. Given the risk of vasospasm inherent to blast-related pTBI, this patient subgroup mandates consideration of distinct management.^35^

## Conclusion

A methodologically rigorous BTF effort, funded by the U.S. Department of Defense, is underway to update 2001 recommendations for the clinical care of pTBI. The vetted, multi-disciplinary workgroup will also develop the first treatment algorithms for pTBI to bridge gaps in published evidence with the practicalities of patient care.^17^ Future work will strategize and examine dissemination and implementation of the finalized guideline and algorithm to assess impact.

## Notes

**Cite this article as:** Hawryluk, GWJ, Selph, S, Lumba-Brown, A, Totten, AM, Ghajar, J, Aarabi, B, Ecklund, J, Shackelford, S, Adams, B, Adelson, D, Armonda, RA, Benjamin, J, Boone, D, Brody, D, Dengler, B, Figaji, A, Grant, G, Harris, O, Hoffer, A, Kitigawa, R, Latham, K, Neal, C, Okonkwo, DO, Pannell, D, Rosenfeld, JV, Rosenthal, G, Rubiano, A, Stein, DM, Stippler, M, Talbot, M, Valadka, A, Wright, DW, Davis, S, Bell, R (2022) Rationale and methods for updated guidelines for the management of penetrating traumatic brain injury. *Neurotrauma Reports* 3:1, 240–247, DOI:10.1089/neur.2022.0008.

## Abbreviations Used

BTF: Brain Trauma Foundation
PICOTS: Population, Interventions, Comparators, Outcomes, Timing, Setting and Study design
pTBI: penetrating traumatic brain injury
SIBICC: Seattle International Severe Traumatic Brain Injury Consensus Conference
TBI: traumatic brain injury

## References

[1] Aarabi, B., Tofighi, B., Kufera, J.A., Hadley, J., Ahn, E.S., Cooper, C., Malik, J.M., Naff, N.J., Chang, L., Radley, M., Kheder, A., and Uscinski, R.H. (2014). Predictors of outcome in civilian gunshot wounds to the head. J. Neurosurg. 120, 1138–1146. 24506239 10.3171/2014.1.JNS131869

[2] Deng, H., Yue, J.K., Winkler, E.A., Dhall, S.S., Manley, G.T., and Tarapore, P.E. (2019). Adult firearm-related traumatic brain injury in United States trauma centers. J. Neurotrauma 36, 322–337. 29855212 10.1089/neu.2017.5591

[3] Larkin, M.B., Graves, E.K.M., Boulter, J.H., Szuflita, N.S., Meyer, R.M., Porambo, M.E., Delaney, J.J., and Bell, R.S. (2018). Two-year mortality and functional outcomes in combat-related penetrating brain injury: battlefield through rehabilitation. Neurosurg. Focus 45, E4. 10.3171/2018.9.FOCUS18359 30544304

[4] Bell, R.S., Mossop, C.M., Dirks, M.S., Stephens, F.L., Mulligan, L., Ecker, R., Neal, C.J., Kumar, A., Tigno, T., and Armonda, R.A. (2010). Early decompressive craniectomy for severe penetrating and closed head injury during wartime. Neurosurg. Focus 28, E1. 10.3171/2010.2.FOCUS1022 20568925

[5] Bell, R.S., Vo, A.H., Neal, C.J., Tigno, J., Roberts, R., Mossop, C., Dunne, J.R., and Armonda, R.A. (2009). Military traumatic brain and spinal column injury: a 5-year study of the impact blast and other military grade weaponry on the central nervous system. J. Trauma 66, S104–S111. 19359953 10.1097/TA.0b013e31819d88c8

[6] DuBose, J.J., Barmparas, G., Inaba, K., Stein, D.M., Scalea, T., Cancio, L.C., Cole, J., Eastridge, B., and Blackbourne, L. (2011). Isolated severe traumatic brain injuries sustained during combat operations: demographics, mortality outcomes, and lessons to be learned from contrasts to civilian counterparts. J. Trauma 70, 11–16; discussion, 16–18. 10.1097/TA.0b013e318207c563 21217475

[7] Ecker, R.D., Mulligan, L.P., Dirks, M., Bell, R.S., Severson, M.A., Howard, R.S., and Armonda, R.A. (2011). Outcomes of 33 patients from the wars in Iraq and Afghanistan undergoing bilateral or bicompartmental craniectomy. J. Neurosurg. 115, 124–129. 21438659 10.3171/2011.2.JNS101490

[8] Rosenfeld, J.V., McFarlane, A.C., Bragge, P., Armonda, R.A., Grimes, J.B., and Ling, G.S. (2013). Blast-related traumatic brain injury. Lancet. Neurol. 12, 882–893. 23884075 10.1016/S1474-4422(13)70161-3

[9] Weisbrod, A.B., Rodriguez, C., Bell, R., Neal, C., Armonda, R., Dorlac, W., Schreiber, M., and Dunne, J.R. (2012). Long-term outcomes of combat casualties sustaining penetrating traumatic brain injury. J. Trauma Acute Care Surg. 73, 1525–1530. 23188247 10.1097/TA.0b013e318270e179

[10] Shackelford, S.A., Del Junco, D.J., Reade, M.C., Bell, R., Becker, T., Gurney, J., McCafferty, R., and Marion, D.W. (2018). Association of time to craniectomy with survival in patients with severe combat-related brain injury. Neurosurg. Focus 45, E2. 10.3171/2018.9.FOCUS18404 30544314

[11] Klimo, P. Jr., and Ragel, B.T. (2010). Introduction: military neurosurgery, past and present. Neurosurg. Focus 28, Introduction. 10.3171/2010.3.FOCUSMar2010Intro 20568950

[12] (2001). Part 1: Guidelines for the management of penetrating brain injury. Introduction and methodology. J. Trauma 51, S3–S6. 11505192

[13] Chesnut, R., Aguilera, S., Buki, A., Bulger, E., Citerio, G., Cooper, D.J., Arrastia, R.D., Diringer, M., Figaji, A., Gao, G., Geocadin, R., Ghajar, J., Harris, O., Hoffer, A., Hutchinson, P., Joseph, M., Kitagawa, R., Manley, G., Mayer, S., Menon, D.K., Meyfroidt, G., Michael, D.B., Oddo, M., Okonkwo, D., Patel, M., Robertson, C., Rosenfeld, J.V., Rubiano, A.M., Sahuquillo, J., Servadei, F., Shutter, L., Stein, D., Stocchetti, N., Taccone, F.S., Timmons, S., Tsai, E., Ullman, J.S., Vespa, P., Videtta, W., Wright, D.W., Zammit, C., and Hawryluk, G.W.J. (2020). A management algorithm for adult patients with both brain oxygen and intracranial pressure monitoring: the Seattle International Severe Traumatic Brain Injury Consensus Conference (SIBICC). Intensive Care Med. 46, 919–929. 31965267 10.1007/s00134-019-05900-x PMC7210240

[14] Hawryluk, G.W.J., Aguilera, S., Buki, A., Bulger, E., Citerio, G., Cooper, D.J., Arrastia, R.D., Diringer, M., Figaji, A., Gao, G., Geocadin, R., Ghajar, J., Harris, O., Hoffer, A., Hutchinson, P., Joseph, M., Kitagawa, R., Manley, G., Mayer, S., Menon, D.K., Meyfroidt, G., Michael, D.B., Oddo, M., Okonkwo, D., Patel, M., Robertson, C., Rosenfeld, J.V., Rubiano, A.M., Sahuquillo, J., Servadei, F., Shutter, L., Stein, D., Stocchetti, N., Taccone, F.S., Timmons, S., Tsai, E., Ullman, J.S., Vespa, P., Videtta, W., Wright, D.W., Zammit, C., and Chesnut, R.M. (2019). A management algorithm for patients with intracranial pressure monitoring: the Seattle International Severe Traumatic Brain Injury Consensus Conference (SIBICC). Intensive Care Med. 45, 1783–1794. 31659383 10.1007/s00134-019-05805-9 PMC6863785

[15] (2000). The Brain Trauma Foundation. The American Association of Neurological Surgeons. The Joint Section on Neurotrauma and Critical Care. Critical pathway for the treatment of established intracranial hypertension. J. Neurotrauma 17, 537–538. 10937898 10.1089/neu.2000.17.537

[16] Bullock, R., Chesnut, R.M., Clifton, G., Ghajar, J., Marion, D.W., Narayan, R.K., Newell, D.W., Pitts, L.H., Rosner, M.J., and Wilberger, J.W. (1996). Guidelines for the management of severe head injury. Brain Trauma Foundation. Eur. J. Emerg. Med. 3, 109–127. 9028756 10.1097/00063110-199606000-00010

[17] Hawryluk, G.W.J., and Ghajar, J. (2021). Evolution and impact of the Brain Trauma Foundation guidelines. Neurosurgery 89, 1148–1156. 34634822 10.1093/neuros/nyab357

[18] Chesnut, R., Aguilera, S., Buki, A., Bulger, E., Citerio, G., Cooper, D.J., Arrastia, R.D., Diringer, M., Figaji, A., Gao, G., Geocadin, R., Ghajar, J., Harris, O., Hoffer, A., Hutchinson, P., Joseph, M., Kitagawa, R., Manley, G., Mayer, S., Menon, D.K., Meyfroidt, G., Michael, D.B., Oddo, M., Okonkwo, D., Patel, M., Robertson, C., Rosenfeld, J.V., Rubiano, A.M., Sahuquillo, J., Servadei, F., Shutter, L., Stein, D., Stocchetti, N., Taccone, F.S., Timmons, S., Tsai, E., Ullman, J.S., Vespa, P., Videtta, W., Wright, D.W., Zammit, C., and Hawryluk, G.W.J. (2020). A management algorithm for adult patients with both brain oxygen and intracranial pressure monitoring: the Seattle International Severe Traumatic Brain Injury Consensus Conference (SIBICC). Intensive Care Med. 46, 919–929. 31965267 10.1007/s00134-019-05900-x PMC7210240

[19] Gerber, L.M., Chiu, Y.L., Carney, N., Hartl, R., and Ghajar, J. (2013). Marked reduction in mortality in patients with severe traumatic brain injury. J. Neurosu.rg 119, 1583–1590. 10.3171/2013.8.JNS13276 24098983

[20] Faul, M., Wald, M.M., Rutland-Brown, W., Sullivent, E.E., and Sattin, R.W. (2007). Using a cost-benefit analysis to estimate outcomes of a clinical treatment guideline: testing the Brain Trauma Foundation guidelines for the treatment of severe traumatic brain injury. J. Trauma 63, 1271–1278. 18212649 10.1097/TA.0b013e3181493080

[21] Brain Trauma Foundation; American Association of Neurological Surgeons; Congress of Neurological Surgeons; Joint Section on Neurotrauma and Critical Care, AANS/CNS; Carney, N.A., and Ghajar, J. (2007). Guidelines for the management of severe traumatic brain injury. Introduction. J. Neurotrauma 24, Suppl. 1, S1–S2. 17511535 10.1089/neu.2007.9997

[22] Carney, N., Totten, A.M., O'Reilly, C., Ullman, J.S., Hawryluk, G.W., Bell, M.J., Bratton, S.L., Chesnut, R., Harris, O.A., Kissoon, N., Rubiano, A.M., Shutter, L., Tasker, R.C., Vavilala, M.S., Wilberger, J., Wright, D.W., and Ghajar, J. (2017). Guidelines for the Management of Severe Traumatic Brain Injury, Fourth Edition. Neurosurgery 80, 6–15. 27654000 10.1227/NEU.0000000000001432

[23] Adelson, P.D., Bratton, S.L., Carney, N.A., Chesnut, R.M., du Coudray, H.E., Goldstein, B., Kochanek, P.M., Miller, H.C., Partington, M.D., Selden, N.R., Warden, C.R., and Wright, D.W.; American Association for Surgery of Trauma; Child Neurology Society; International Society for Pediatric Neurosurgery; International Trauma Anesthesia and Critical Care Society; Society of Critical Care Medicine; World Federation of Pediatric Intensive and Critical Care Societies. (2003). Guidelines for the acute medical management of severe traumatic brain injury in infants, children, and adolescents. Chapter 11. Use of hyperosmolar therapy in the management of severe pediatric traumatic brain injury. Pediatr. Crit. Care Med. 4, S40–S44. 12847347

[24] Badjatia, N., Carney, N., Crocco, T.J., Fallat, M.E., Hennes, H.M., Jagoda, A.S., Jernigan, S., Letarte, P.B., Lerner, E.B., Moriarty, T.M., Pons, P.T., Sasser, S., Scalea, T., Schleien, C.L., and Wright, D.W.; Brain Trauma Foundation; BTF Center for Guidelines Management. (2008). Guidelines for prehospital management of traumatic brain injury 2nd edition. Prehosp. Emerg. Care 12, Suppl. 1, S1–S52. 18203044 10.1080/10903120701732052

[25] (2000). The Brain Trauma Foundation. The American Association of Neurological Surgeons. The Joint Section on Neurotrauma and Critical Care. Age. J. Neurotrauma 17, 573–581. 10937903 10.1089/neu.2000.17.573

[26] Hawryluk, G.W.J., Aguilera, S., Buki, A., Bulger, E., Citerio, G., Cooper, D.J., Arrastia, R.D., Diringer, M., Figaji, A., Gao, G., Geocadin, R., Ghajar, J., Harris, O., Hoffer, A., Hutchinson, P., Joseph, M., Kitagawa, R., Manley, G., Mayer, S., Menon, D.K., Meyfroidt, G., Michael, D.B., Oddo, M., Okonkwo, D., Patel, M., Robertson, C., Rosenfeld, J.V., Rubiano, A.M., Sahuquillo, J., Servadei, F., Shutter, L., Stein, D., Stocchetti, N., Taccone, F.S., Timmons, S., Tsai, E., Ullman, J.S., Vespa, P., Videtta, W., Wright, D.W., Zammit, C., and Chesnut, R.M. (2019). A management algorithm for patients with intracranial pressure monitoring: the Seattle International Severe Traumatic Brain Injury Consensus Conference (SIBICC). Intensive Care Med. 45, 1783–1794. 31659383 10.1007/s00134-019-05805-9 PMC6863785

[27] (2001). Neuroimaging in the management of penetrating brain injury. J. Trauma 51, S7–S11. 11505193

[28] (2001). Intracranial pressure monitoring in the management of penetrating brain injury. J. Trauma 51, S12–S15. 11505194

[29] (2001). Surgical management of penetrating brain injury. J. Trauma 51, 2 Suppl., S16–S25. 11505195

[30] (2001). Vascular complications of penetrating brain injury. J. Trauma 51, 2 Suppl., S26–S28. 11505196

[31] (2001). Management of cerebrospinal fluid leaks. J. Trauma 51, 2 Suppl., S29–S33. 11505197

[32] (2001). Antibiotic prophylaxis for penetrating brain injury. J. Trauma 51, 2 Suppl., S34–S40. 11505198

[33] (2001). Antiseizure prophylaxis for penetrating brain injury. J. Trauma 51, 2 Suppl., S41–S43. 11505199

[34] Bell, R.S., Ecker, R.D., Severson, M.A. III, Wanebo, J.E., Crandall, B., and Armonda, R.A. (2010). The evolution of the treatment of traumatic cerebrovascular injury during wartime. Neurosurg. Focus 28, E5. 10.3171/2010.2.FOCUS1025 20568945

[35] Rish, B.L., Dillon, J.D., Caveness, W.F., Mohr, J.P., Kistler, J.P., and Weiss, G.H. (1980). Evolution of craniotomy as a debridement technique for penetrating craniocerebral injuries. J. Neurosurg. 53, 772–775. 7441337 10.3171/jns.1980.53.6.0772

[36] Carey, M.E., Young, H., Mathis, J.L., and Forsythe, J. (1971). A bacteriological study of craniocerebral missile wounds from Vietnam. J. Neurosurg. 34, 145–154. 14768680 10.3171/jns.1971.34.2part1.0145

[37] Hagan, R.E. (1971). Early complications following penetrating wounds of the brain. J. Neurosurg. 34, 132–141. 14768678 10.3171/jns.1971.34.2part1.0132

[38] Hammon, W.M. (1971). Analysis of 2187 consecutive penetrating wounds of the brain from Vietnam. J. Neurosurg. 34, 127–131. 14768677 10.3171/jns.1971.34.2part1.0127

[39] Rosenfeld, J.V., Bell, R.S., and Armonda, R. (2015). Current concepts in penetrating and blast injury to the central nervous system. World J. Surg. 39, 1352–1362. 25446474 10.1007/s00268-014-2874-7 PMC4422853

[40] Bell, R.S., Vo, A.H., Roberts, R., Wanebo, J., and Armonda, R.A. (2010). Wartime traumatic aneurysms: acute presentation, diagnosis, and multimodal treatment of 64 craniocervical arterial injuries. Neurosurgery 66, 66–79; discussion, 79. 20023539 10.1227/01.NEU.0000361285.50218.A8

[41] Armonda, R.A., Bell, R.S., Vo, A.H., Ling, G., DeGraba, T.J., Crandall, B., Ecklund, J., and Campbell, W.W. (2006). Wartime traumatic cerebral vasospasm: recent review of combat casualties. Neurosurgery 59, 1215–1225; discussion, 1225. 17277684 10.1227/01.NEU.0000249190.46033.94

[42] Centers for Disease Control and Prevention. (2022). Explosions and Blast Injuries: A Primer for Clinicians. United States Department of Health and Human Services. Available from: https://www.cdc.gov/masstrauma/preparedness/primer.pdf [Las accessed May 26, 2022].

